# Epidemiological and Histopathological Investigation of *Sarcocystis* spp. in Slaughtered Dromedary Camels (*Camelus dromedarius*) in Egypt

**DOI:** 10.3390/vetsci7040162

**Published:** 2020-10-27

**Authors:** Ahmed Gareh, Mahmoud Soliman, Amira A. Saleh, Fatma A. El-Gohary, Heba M. M. El-Sherbiny, Ragab H. Mohamed, Ehab Kotb Elmahallawy

**Affiliations:** 1 Department of Parasitology, Faculty of Veterinary Medicine, Aswan University, Aswan 24101, Egypt; ahmedgareh86@gmail.com; 2 Department of Pathology and Clinical Pathology, Faculty of Veterinary Medicine, Assiut University, Assiut 71515, Egypt; selemanvet08@yahoo.com; 3 Department of Medical Parasitology, Faculty of Medicine, Zagazig University, Zagazig 44519, Egypt; Amera.islam2011@yahoo.com; 4 Department of Hygiene and Zoonoses, Faculty of Veterinary Medicine, Mansoura University, Mansoura 35516, Egypt; dr.fatmagohary@gmail.com; 5 Educational Veterinary Hospital, Faculty of Veterinary Medicine, Mansoura University, Mansoura 35516, Egypt; hebaelsherbiny11@gmail.com; 6 Department of Theriogenology, Faculty of Veterinary Medicine, Aswan University, Aswan 24101, Egypt; ragabhasan2016@gmail.com; 7 Department of Biomedical Sciences, University of León (ULE), 24071 León, Spain; 8 Department of Zoonoses, Faculty of Veterinary Medicine, Sohag University, Sohag 82524, Egypt

**Keywords:** camel, Egypt, epidemiology, histopathology, *Sarcocystis* spp.

## Abstract

Sarcocystosis is considered one of the major parasitic diseases with a worldwide distribution. It is caused by the obligatory intracellular protozoan parasites of the genus *Sarcocystis*. Besides its public health issues, sarcocystosis results in significant economic losses due to its impact on productivity and milk yield. A wide range of final and intermediate hosts have been identified, including mammals, birds, and reptiles; however, few studies have investigated the contribution of camels to maintaining the epidemiological foci of the disease in countries such as Egypt. The present study was conducted to grossly and histopathologically identify the prevalence rate of *Sarcocystis* spp. in camels (*N* = 100) from the Aswan Governorate, Egypt. Furthermore, the major risk factors related to the development of sarcocystosis in camels were investigated. Samples from the diaphragm, cardiac muscle, esophagus, and testes of the slaughtered camels were collected. Interestingly, *Sarcocystis* was detected in 75% of the examined camels. Following the studied variable factors, camels aged 5 years or more were found to be at higher risk, with an infection rate of 87.7% (57 of 65) than those younger than 5 years. The infection rate was 81.4% (57 of 70) in males and 60% (18 of 30) in females. The esophagus was the most affected organ (49%), followed by the diaphragm (26%) and cardiac muscle (17%), whereas none of the testes samples were affected. Taken together, the present study demonstrates the high prevalence of *Sarcocystis* in the examined camels and suggests the importance of these animals in preserving the epidemiological foci of sarcocystosis in Egypt. Future research should map the circulating strains in Egypt and aim to raise public health awareness about the importance of sarcocystosis and other related zoonotic diseases.

## 1. Introduction

*Sarcocystis* spp. are intracellular protozoan parasites of the phylum Apicomplexa. They are considered one of the most common parasites with global distribution in humans and various animal species [1,2,3]. The parasite can infect a variety of intermediate hosts, including mammals, birds, and reptiles, whereas carnivores act as the final hosts [2,3,4,5,6,7]. The final hosts contract the infection by ingestion of muscle cysts containing bradyzoites, whereas schizonts and merozoites are not infective for the final hosts [8]. Camels may serve as intermediate hosts and can develop an infection following the ingestion of sporulated oocysts excreted in feces from infected carnivores. In the camel gut, sporozoites excyst, divide rapidly in the gut wall, and then migrate to the skeletal and cardiac muscles to produce the distinctive sarcocyst. The life cycle then terminates when infective sarcocysts are ingested by the final host, which is typically a member of the Canidae family in the case of *Sarcocystis cameli* [6,9,10]. Sporulated oocysts undergo asexual and sexual reproduction and are passed in the feces of final hosts [6]. Sarcocysts are located mainly in the skeletal and cardiac muscles, occasionally in the brain. The size of these cysts varies by species and ranges from a few millimeters to centimeters in length [11].

Note that *Sarcocystis* spp. include macroscopic and microscopic species; however, macroscopic species are not important pathogenic agents when compared with microscopic ones, which comprise some zoonotic species. The microscopic species cannot be identified through routine meat inspection and therefore do not lead to carcass condemnation. However, the high prevalence of these microscopic forms has a high economic impact on animal production [6,12,13]. The first study of *Sarcocystis* infection in camels (*Camelus dromedarius*) from Egypt was reported in 1910; the observed sarcocysts were less than 12 mm in length, 1 mm in width, and appeared to be white lines with thin or thick cyst walls [14]. Clearly, at least two morphologically different *Sarcocystis* were affecting the camels (thin- and thick-walled) [15,16,17].

Humans acquire the infection through the ingestion of undercooked meat of animals contaminated with certain species of *Sarcocystis* [6]. Humans are the final hosts of *Sarcocystis hominis* and *Sarcocystis suihominis*, with cattle and pigs as intermediate hosts, respectively [18]. Given the predator–prey relationship of the parasite, sarcocystosis does not represent a serious health threat to humans who might serve as the dead-end hosts [1,19]. Hence, the disease is often asymptomatic in definitive hosts [1,19,20]. In humans, the infection may lead to two different scenarios. In the first one, intestinal infection is usually caused by two species of coccidian parasites, namely, *S. hominis* and *S. suihominis*, developed through the consumption of raw infected beef and pork, respectively. The resulting symptoms may include nausea, vomiting, stomachache, diarrhea, and dyspnea. The second scenario is muscular involvement, which happens when humans serve as intermediate hosts. This presentation may be associated with muscle pain, transitory edema, and fever [6,21,22]. However, no reports of the transmission of *S. cameli* to humans through the consumption of camel meat are available. Clinical manifestations of the acute form of sarcocystosis in animal intermediate hosts may include encephalitis, bleeding diathesis, and inflammation of the brain and spinal cord [20,23]. The disease may also result in death, premature delivery, or abortion in pregnant animals [19,24]. Meanwhile, mild and chronic sarcocystosis leads to a drop in body weight and fur count, as well as significant changes in animal behavior [5]. Collectively, the parasite can affect animal growth and weight gain; reduce meat quality and milk yield; and cause anorexia, fever, anemia, abortion, muscle weakness, and even death of the intermediate hosts [4].

Importantly, mapping the epidemiology of these parasites is one of the key strategies for controlling this disease, and epidemiological data can inform efforts to intercept the life cycle. It is often very difficult to diagnose the acute stage of the disease in intermediate hosts. Various diagnostic methods are available for the detection of sarcocystosis, which include muscle squashing, pepsin digestion, and histological techniques [25]. Histopathological examination of samples offers many advantages in the detection of *Sarcocystis* in the major host groups [4,7,26]. Furthermore, the exploration of various epidemiological variables and risk factors through field surveys appears to be critical to the implementation of effective intervention strategies besides raising public health awareness of the disease. Given the scarcity of information on sarcocystosis in camels in countries such as Egypt, the present study was undertaken to identify the prevalence of *Sarcocystis* infection in camels in the Aswan Governorate (Egypt) by examining histopathological changes and estimating the major variable risk factors potentially associated with the infection.

## 2. Materials and Methods

### 2.1. Ethics Statement

The study protocol was carefully reviewed and approved by the local guidance of Research, Publication, and Ethics of the Faculty of Veterinary Medicine, Mansoura University, Egypt, which complies with all the relevant Egyptian laws.

### 2.2. Sampling and Study Area

The present study included 100 camels. Animals were slaughtered in the abattoir of Daraw, Aswan Governorate (Egypt), from March to November 2019. The Aswan Governorate (Egypt) is the southernmost part of Upper Egypt (latitude, 24°5′20.1768″ N and longitude 32°53′59.3880″ E) and is located near the borders of Sudan. This province has a very strategic location for the importation of animals from other African countries.

### 2.3. Macroscopic Examination

Camels were admitted to Daraw abattoir for slaughter. The skeletal muscles of the fore and hind limbs, diaphragm, intercostal muscles, heart, tongue, esophagus, and masseter muscles were carefully inspected by the naked eye for the presence of *Sarcocystis*, as described elsewhere [4].

### 2.4. Microscopic Examination

Microscopic examination was conducted as follows:

**(A)**  **Direct examination:** Small tissue samples (2 mm × 8 mm) from the cardiac muscle, esophagus, diaphragm, and testes were squashed between two glass slides and examined by light microscopy (×100). Later on, two preparations were made from each muscle sample and examined for the positivity of *Sarcocystis*, as described elsewhere [27].
**(B)**  **Digestion method:** Tissue digestion was used for enabling the observation of bradyzoites in the tested organ samples. Seventy grams of each tissue sample was ground and digested in 1.5% HCL acid and 0.5% pepsin at 29 °C overnight. The digested samples were then filtered through a mesh and centrifuged at 1500 rpm for 10 min. Next, the supernatant fluid was discarded. The sediment was stained with Giemsa and examined microscopically for the detection of bradyzoites [28].
**(C)**  **Histopathological examination:** Tissue specimens from positive cases were fixed in 10% neutral buffered formalin, dehydrated in graded alcohol series, cleared in xylene, embedded in paraffin, sliced into 5 μm thick sections, and mounted on slides. The slides were then stained with hematoxylin and eosin and were examined microscopically [29].

### 2.5. Statistical Analysis

Statistical analysis was performed using the statistical software SPSS (Version 22, SPSS Inc., Chicago, MI, USA) for Windows, and chi-square (χ^2^) tests were used to evaluate the correlation between the occurrence of *Sarcocystis* spp. and major variable risk factors for infection (age, sex, and organ involved). A probability (*p*) value of <0.05 was considered to indicate statistical significance.

## 3. Results

### 3.1. Prevalence of Sarcocystis spp. by Macroscopic and Direct Examination Methods

Note that *Sarcocystis* could not be detected macroscopically during the postmortem examination of the slaughtered camels. Table 1 shows the prevalence of infection with *Sarcocystis* spp. and associated risk factors. The prevalence of microscopic *Sarcocystis* by direct examination in examined camels was 75% (75 of 100).

### 3.2. Prevalence of Sarcocystis spp. by the Tissue Digestion Method

The tissue digestion method was used to verify the results of the prevalence obtained by direct examination. Similarly to direct examination, *Sarcocystis* could be detected in 75% of examined camels using the tissue digestion method.

### 3.3. Occurrence of Sarcocystis spp. in Examined Camels and Major Risk Factors Associated with Infection 

The age, sex, and infected organ of the animals were all significantly associated with *Sarcocystis* infection. In this regard, camels aged 5 years or older were found to be at higher risk of the infection (infection rate, 87.7% (57 of 65)) than those younger than 5 years (infection rate, 51.4% (18 of 35)). Male animals were more affected than female animals (81.4% (57of 70) versus 60% (18 of 30)). Our data showed that the esophagus was the most affected organ with an infection rate of 49%; the diaphragm and cardiac muscle were infected by 26% and 17%, respectively, whereas no sarcocysts were detected in testes samples (Table 1).

### 3.4. Histopathological Findings

**Figure 1:**
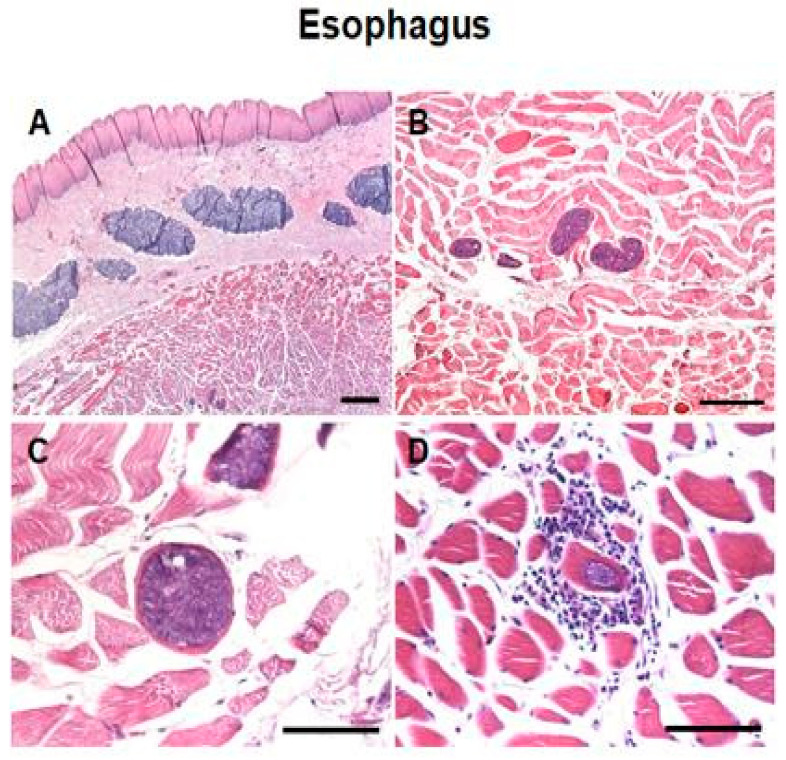
Photomicrograph of *Sarcocystis* in the esophagus. Hematoxylin and eosin stain. Encysted parasites in the muscle fibers of the esophagus without (**A**–**C**) or with (**D**) inflammatory cells infiltration. Bar (**A**) = 500 µM. Bar (**B**) = 100 µM. Bars (**C**,**D**) = 50 µM.

Irregularly shaped *Sarcocystis* were observed within the muscle fibers of the oesophagus, diaphragm and heart (Figure 1A–C and Figures S1 and S2). Cysts contained several basophilic bradyzoites. Generally, there were no degenerative or inflammatory changes in the infected tissues; however, we observed the infiltration of inflammatory cells around a single sarcocyst in an esophageal muscle sample (Figure 1D).

## 4. Discussion

As previously mentioned, *Sarcocystis* spp. are a group of coccidian parasites that infect warm-blooded animals, including humans who might act as final or intermediate hosts [2,30]. The disease is mostly self-limiting in humans; however, several outbreaks of intestinal and muscular sarcocystosis were reported. A review of the available literature shows that approximately 150–200 species of *Sarcocystis* have been identified and described in a wide range of hosts based on parasite morphology; however, the exact number of species remains unidentified [2,31]. Importantly, *Sarcocystis* spp. are among the most common and widespread protozoan parasites of veterinary and economic importance [6,32].

The present study provides interesting data related to the occurrence of *Sarcocystis* spp. in camels from the Aswan Governorate, Egypt. To this end, our study revealed the major risk factors associated with the infection in these camels. We were unable to identify any cases of macroscopic *Sarcocystis*-associated pathology during the postmortem examination of slaughtered camels, which is in agreement with previous reports from Iran and Egypt [4,26,33,34]. Prevalence data on *Sarcocystis* species in camels have been reported in various countries, including Sudan, Jordan, Kazakhstan, Afghanistan, Morocco, the former Union of Soviet Socialist Republics [6,17], Egypt [35], Somalia [36], Saudi Arabia [15], Iraq [37], Southern Ethiopia [38], and Mongolia [27].

As shown in the present study, the prevalence of *Sarcocystis* infection in slaughtered camels in the Aswan Governorate using microscopic examination was 75%. The observed prevalence is higher than that reported in Egypt, Southern Ethiopia, and the Yazd Province (Iran), where the reported prevalence rates were 42.3–60.0%, 45.45%, and 51.5%, respectively [33,34,38,39]. Moreover, a couple of previous reports on *Sarcocystis* spp. in camels from Saudi Arabia, Afghanistan, and Morocco reported prevalence rates of 56.7%, 47.3–66.3%, and 60%, respectively [40,41,42]. In a previous study in Jordan, the reported prevalence was 6.6%, which is markedly lower than that identified in the present study [43]. In stark contrast, higher infection rates of 100% and 91.6% were reported in Mongolia and Iraq, respectively [27,37]. Furthermore, in a previous study in Egypt, a prevalence of 81% was reported [44], whereas a prevalence of 83.6% was reported in the eastern Provinces of Iran [4]. The variation between our present results and those previously mentioned could be attributed to various factors including the degree of contact between camels and dogs since some camel pastoralists are not using dogs in camel rearing (camels are reared on a free-range basis in the desert); therefore, differences in the systems used for camel keeping could influence the infection rate [4,7]. Meanwhile, a high prevalence (78.9%) of *Sarcocystis* infection was reported among slaughtered buffaloes in Beni-Suef, Egypt, and the authors attributed the high prevalence in this region to the close rearing of buffaloes with dogs, cats, and even wild animals that act as final hosts for these protozoa [45].

In the present study, age was found to be a significant variable associated with infection. Older camels appeared to be at greater risk of acquiring infection than younger ones; hence, animals aged 5 years or older were infected to a significantly larger extent (87.7%) than younger animals (51.4%) χ^2^ = 15.956, *p* = 0.000). Similar findings were reported in previous studies in the Menofia Governorate (Egypt) [46], Yazd Province (Iran) [33], and in Riyadh city (Saudi Arabia) [47]. The higher prevalence of *Sarcocystis* infection in aged camels may likely reflect the higher rate of slaughtering of aged camels compared with younger animals; moreover, slow development of detectable cysts may explain the lower prevalence in young camels [4,33,47]. Additionally, some owners kept the young camels indoor for breeding, and therefore, the young camels might be less exposed to infection than older ones [4,33].

In the present investigation, the sex of the animal was another significant risk factor, with males being at higher risk of infection than females (χ^2^ = 5.143, *P* = 0.023). As shown in Table 1, prevalence rates of 81.4% and 60% were reported in male and female camels, respectively. Our finding is consistent with several previous studies in Egypt [34], southern Ethiopia [38], and Iran [4]. This difference might be attributed to the fact that most female animals are kept indoor for reproduction under good and clean management, whereas most of the males are left for grazing outdoor and used by owners for hard work; they may therefore be more exposed to the infection [48].

*Sarcocystis* spp. infect muscular tissue of the heart, tongue, esophagus, and diaphragm. However, *Sarcocystis* spp. cysts have been reported in several other types of muscle tissue [49,50]. Furthermore, some studies reported sarcocystosis in the cremaster muscle of an animal with orchitis, which observation encouraged us to investigate sarcocysts in testicular tissue samples [49,51]. In the present study, dissemination of *Sarcocystis* in different organs was observed especially in the esophagus, with a prevalence rate of 49% which is according to several previous reports either in the same species or different species [4,26,33,38,52]. Meanwhile, some studies found the diaphragm of camels to be the most commonly affected site [27,43], whereas another study identified the heart as the most commonly infected organ [53]. However, in other species, such as the water buffalo, the predilection sites for *Sarcocystis* spp. appear to be the esophagus, tongue, and heart [54]. This difference could be explained by different *S. cameli* strains or differences in definite host species [33].

Over the last few decades, extraordinary progress was made in developing the criteria for the identification and diagnosis of sarcocystosis in both intermediate and definitive hosts. Several methods were used to evaluate the sarcocyst and sporocyst morphology in their major intermediate hosts using light microscopy to identify the asexual stages in the infected tissues of intermediate hosts or the sexual stages in the gut of the definitive host. In this regard, the digestion method and histopathological analysis have been implemented for the analysis of microscopic sarcocysts in camels. Moreover, ultrastructural analysis, using transmission electron microscopy of the cyst wall appears very useful in the identification of sarcocysts [55]. Furthermore, several serological techniques, including enzyme-linked immunosorbent assay and an indirect fluorescent antibody test, were used for diagnosing infection. However, these methods are limited by low sensitivity and specificity due to cross-reactivity between various *Sarcocystis* spp. [5,56]. Additionally, the use of molecular methods, such as conventional polymerase chain reaction and restriction fragment length polymorphism, represents an essential alternative accurate technique for the identification of endogenous and exogenous stages of *Sarcocystis* spp. [2,20,57]. However, these methods are typically more expensive compared with microscopic examination, and the lack of financial support remains among the major limitations of using such techniques in the field. Detection of the sporocysts of *Sarcocystis* spp. in the feces of definitive hosts could play an essential diagnostic role; however, based on morphological identification, the various species are very difficult to distinguish [58]. Sarcocysts may take years to grow in size to become macroscopically visible. This fact justifies our inability to detect macroscopic sarcocysts in our present study. On the one hand, microscopic evaluation of muscle tissue samples is important in the diagnosis of *Sarcocystis* infections in camels. On the other hand, detection of the parasite stages (sarcocysts) in tissues using histopathological methods provides a confirmatory tool for the diagnosis [2]. In the present study, histological examination revealed the presence of two morphologically distinct *Sarcocystis* embedded within the muscle fibers of the esophagus, diaphragm, and heart. Thin-walled *Sarcocystis* were found to be most common, which is consistent with some previous reports [2]. The morphology of *Sarcocystis* is unique as they are intramyofiber protozoal cysts with two types of cyst walls, a palisade-like thick wall or a smooth thin wall. As shown in Figure 1 and Figures S1 and S2, sarcocysts appear dark blue-colored due to the presence of many crescent-like bradyzoites inside the cysts.

No inflammatory reaction was observed in the tissue surrounding the cysts. The apparent lack of inflammatory response might be attributed to the fact that protozoa are located in cysts within the muscle fibers, which offers protection from host immunity—a hypothesis that has been confirmed for various parasites [59,60,61,62]. Our results are in line with the fact that inflammatory cells are not often reported in *Sarcocystis*-infected tissue [63,64,65].

## 5. Conclusions

Current epidemiological and histopathological findings suggest a high occurrence of *Sarcocystis* in camels in this region of Egypt and indicate that camels may be critical to preserving the epidemiological foci of the disease. The present study also disclosed various major risk factors associated with infection, including animal age, sex, and anatomical predilection site. Further future molecular and epidemiological studies should focus on identifying the major circulating strains in Egyptian ecological niches. Strict hygienic measures may be critical to controlling the disease.

## Figures and Tables

**Table 1:** Prevalence of *Sarcocystis* infection in camels slaughtered at Aswan Governorate.

Variable Categories	Number of Examined Camels	Number of Infected Camels	Proportion of Infected Camels (%)	Chi-Square (χ^2^)	*p* Value
Overall infection	100	75	75		
Age group					
Young (<5 years)	35	18	51.4	15.956	0.000
Aged (≥5 years)	65	57	87.7		
Sex of animal					
Male	70	57	81.4	5.143	0.023
Female	30	18	60		
Affected organ					
Esophagus	100	49	49	57.725	0.000
Diaphragm	100	26	26		
Heart	100	17	17		
Testes	70	0	0.0		

## Supplementary Materials

The following are available online at https://www.mdpi.com/2306-7381/7/4/162/s1, Figure S1: Photomicrograph of Sarcocystis in the diaphragm. Encysted parasites in the muscle fibers of the diaphragm without inflammatory cells infiltration (A–D). Hematoxylin and eosin stain. Bar A = 100 µM. Bars B–D = 50 µM; Figure S2: Photomicrograph of *Sarcocystis* in the heart. Encysted parasite in the muscle fibers of the heart without inflammatory cells infiltration (A and B). Hematoxylin and eosin stain. Bar A = 100 µM. Bar B = 50 µM.
