# Supplementary material for: Epidemiological and Histopathological Investigation of Sarcocystis spp. in Slaughtered Dromedary Camels (Camelus dromedarius) in Egypt
Source: Vet Sci. 2020 Oct 27;7(4):162. doi: 10.3390/vetsci7040162 (PMC7711966; doi:10.3390/vetsci7040162)
Supplement: Supplementary file 1 [file vetsci-07-00162-s001.pdf]

Supplementary

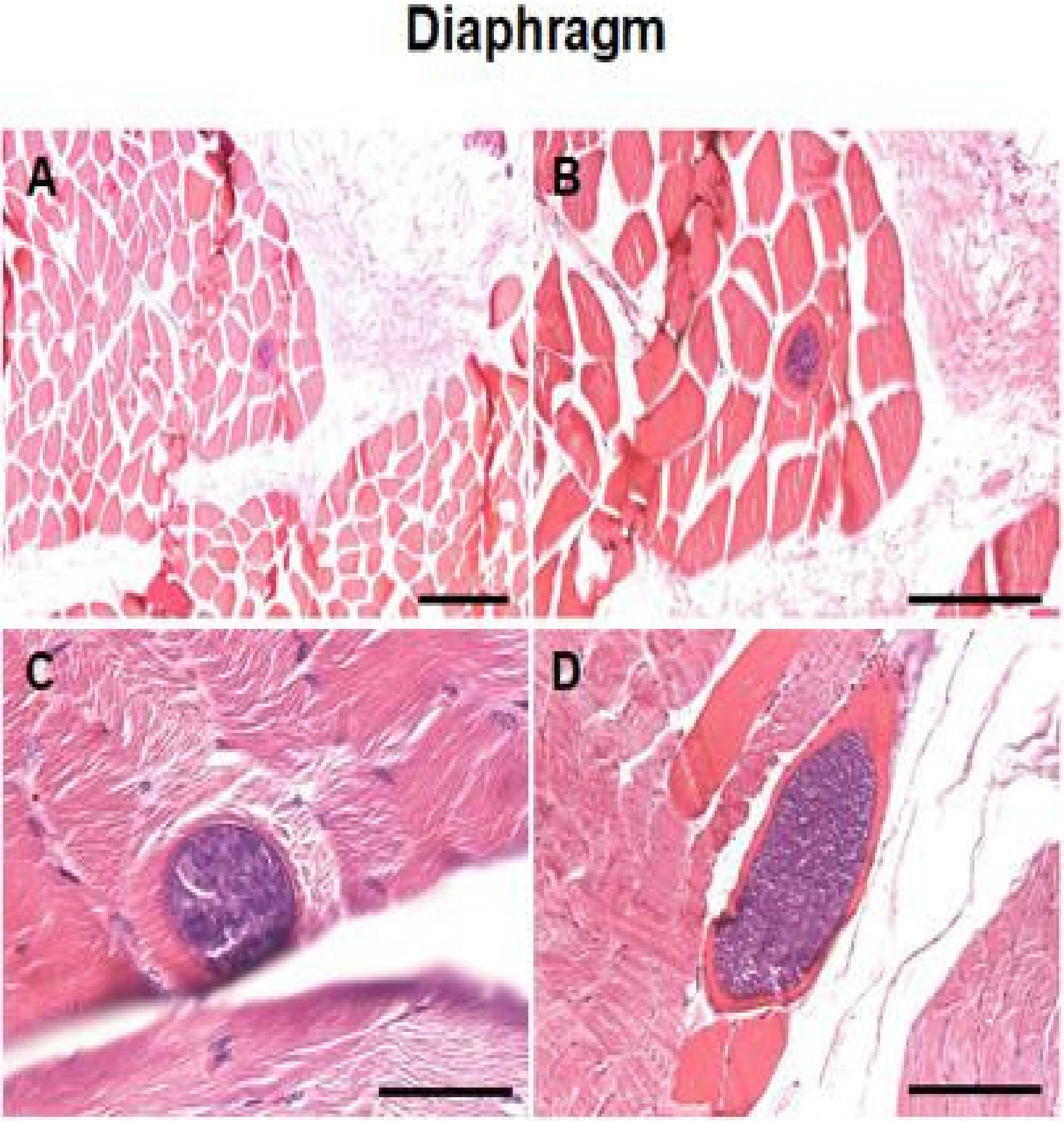

Figure S1. Photomicrograph of *Sarcocystis* in the diaphragm. Encysted parasites in the muscle fibers of the diaphragm without inflammatory cells infiltration (A-D). Hematoxylin and eosin stain. Bar A = 100 μM. Bars B-D = 50 μM.

Heart

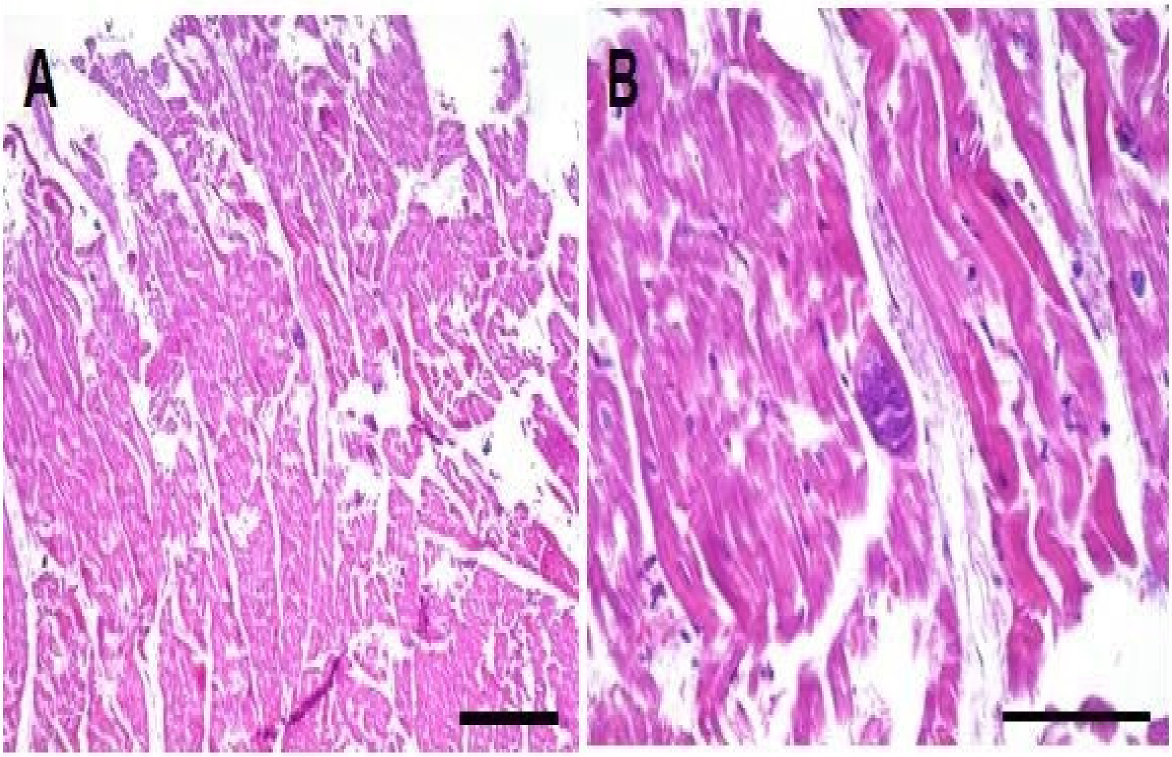

Figure S2. Photomicrograph of *Sarcocystis* in the heart. Encysted parasite in the muscle fibers of the heart without inflammatory cells infiltration (A and B). Hematoxylin and eosin stain. Bar A = 100 µM. Bar B = 50 µM.
